# Global trends in research on extracorporeal shock wave therapy (ESWT) from 2000 to 2021

**DOI:** 10.1186/s12891-023-06407-9

**Published:** 2023-04-20

**Authors:** Xinyu Zhang, Yuewen Ma

**Affiliations:** Department of Rehabilitation, The First Affiliated Hospital of China Medical University, 155 Nanjing Street, 110001 Shenyang, P.R. China

**Keywords:** Extracorporeal shock wave therapy (ESWT), Research hotspots, VOSviewer

## Abstract

**Background:**

This study intended to analyze the application of extracorporeal shock wave therapy in medicine and to evaluate the quality of related literature.

**Methods:**

All publications were extracted from 2000 to 2021 from the Web of Science Core Collection (WoSCC). The literature characteristics were depicted by VOSviewer (version 1.6.15) and the online bibliometric website (http://bibliometric.com/). The future trends and hotspots were conducted by Bibliographic Item Co-occurrence Matrix Builder (version 2.0) and gCLUTO software.

**Results:**

We analyzed 1774 articles corresponding to the criteria for ESWT publications from 2000 to 2021. Most studies were conducted within the United States and China which besides have the most cooperation. The most published research institutions are Chang Gung University, Kaohsiung Chang Gung Memorial Hospital, and Kaohsiung Medical University. Six research hotspots were identified by keyword clustering analysis: Cluster0: The effects of ESWT on muscle spasticity; Cluster1: The application of ESWT in osteoarthritis (OA); Cluster2: Therapeutic effect of ESWT on tendon diseases; Cluster3: Early application of ESWT/ESWL in urolithiasis; Cluster4: The Role of angiogenesis in ESWT and the efficiency of ESWT for penile disease; Cluster5: The Special value of radial extracorporeal shock wave therapy (rESWT).

**Conclusions:**

A comprehensive and systematic bibliometric analysis of ESWT was conducted in our study. We identified six ESWT-related research hotspots and predicted future research trends. With the gradual increase of research on ESWT, we find that ESWT is used more and more extensively, such in musculoskeletal disease, bone delay union, neurological injury, andrology disorders, lymphedema, and so on. In addition, the mechanism is not destructive damage, as initially thought, but a restorative treatment. Furthermore, delayed union, cellulite, burn, and diabetic foot ulcers may be the future direction of scientific study.

## Introduction

Extracorporeal shock wave therapy (ESWT) as a vital treatment in the rehabilitation department, is a kind of mechanical wave with both acoustic and force characteristics [1]. Initially, it was discovered from the treatment of urological extracorporeal lithotripsy [2]. Because of its economy and effectiveness, it has gradually become a very essential means of rehabilitation medicine in recent years. ESWT has been used to treat a variety of conditions, including musculoskeletal injuries, such as plantar fasciitis [3] and tennis elbow [4, 5], as well as some neurological conditions [6, 7]. The technology has developed rapidly in recent years, with the introduction of more advanced equipment and improved understanding of the underlying mechanisms of action.

Bibliometrics is a novel method in literature analysis, which can quantitatively analyze the published literature in co-authorship, keyword co-occurrence, and citation based on the bibliometric data [8]. Bibliometrics is more intuitive and effective than traditional literature analysis methods, which can only be qualitatively summarized by researchers themselves [9]. With great therapeutic progress made by ESWT in the field of rehabilitation medicine, the number of relevant papers has been increasing annually. Few researchers have systematically described and summarized the ESWT domain using bibliometrics so far. In recent years, only one bibliometric study on shock wave therapy (SWT) has been published [10], but it did not provide a detailed discussion on identifying the research hotspots. This study aims to more accurately identify the current research hotspots in ESWT, and to provide an in-depth discussion on the status, trends, and future challenges of these identified hotspots, with the goal of providing researchers with a clearer and more intuitive overview of ESWT. Therefore, we conducted a systematic and comprehensive bibliometrics analysis to evaluate bibliometrics indicators, identify research hotspots and predict future research trends in the ESWT research field. In this study, we [1] identified the most influential authors and institutions, in the field of ESWT, as well as the most highly cited journals in the field. [2] evaluated the impact of the research by examining the number of citations received by papers on the topic and the h-index, which is a measure of the productivity and impact of an author. [3] assessed the research trends and guided future research.

## Materials and methods

### Data acquisition and search strategy

The WoSCC is one of the most authoritative scientific databases, providing both qualitative and quantitative academic information on a global scale. In this study, all documents were retrieved, screened, and downloaded from the WoSCC database. The advanced search query was used: **TS = (ESWT OR extracorporeal shock wave therapy)**.

### Screening criteria and data downloads

The literature is qualified to meet the following requirements: 1) The publication language is English. 2)The range of publication years is between 2000 and 2021. 3)Only articles and reviews can be included. On January 17, 2022, two independent researchers conducted literature retrieval and data download and collation individually. Our agreement rate is up to 99%, demonstrating accuracy [11].

### Statistical analysis

Data were analyzed from the following features, such as countries, institutions, journals, and keywords, and then visualized in the form of figures or tables. We used the VOSviewer (version 1.6.15) and an online platform for literature metrology analysis (http://bibliometric.com/) to conduct the visual analysis. The analysis based on the dataset can be ocular to explore various kinds of potential connections hidden in the text data and offer a kind of brand-new way to scientists. VOSviewer was used to visualize the networks such as institutions, journals, and keywords [12]. We performed the international links between countries (or regions) by the bibliometrics online analysis website. Bibliographic item co-occurrence matrix builder (BICOMB) can convert the literature imported from WoSCC into a binary keyword-article matrix [13]. At last, the graphical clustering toolkit (gCLUTO) (version 1.0) software was applied to cluster the keywords and plot them into mountain maps and heat maps [13]. After all, clustering can strengthen graphs’ visibility and contribute to visual analysis, and observation.

## Results

### Distribution of annual publications

**Fig. 1:**
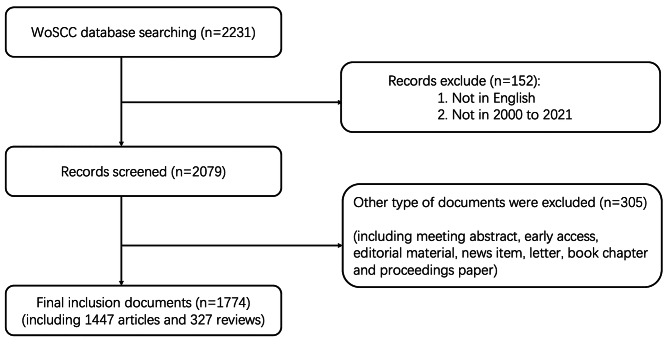
Flowchart of data filtration processing and excluding publications

**Fig. 2:**
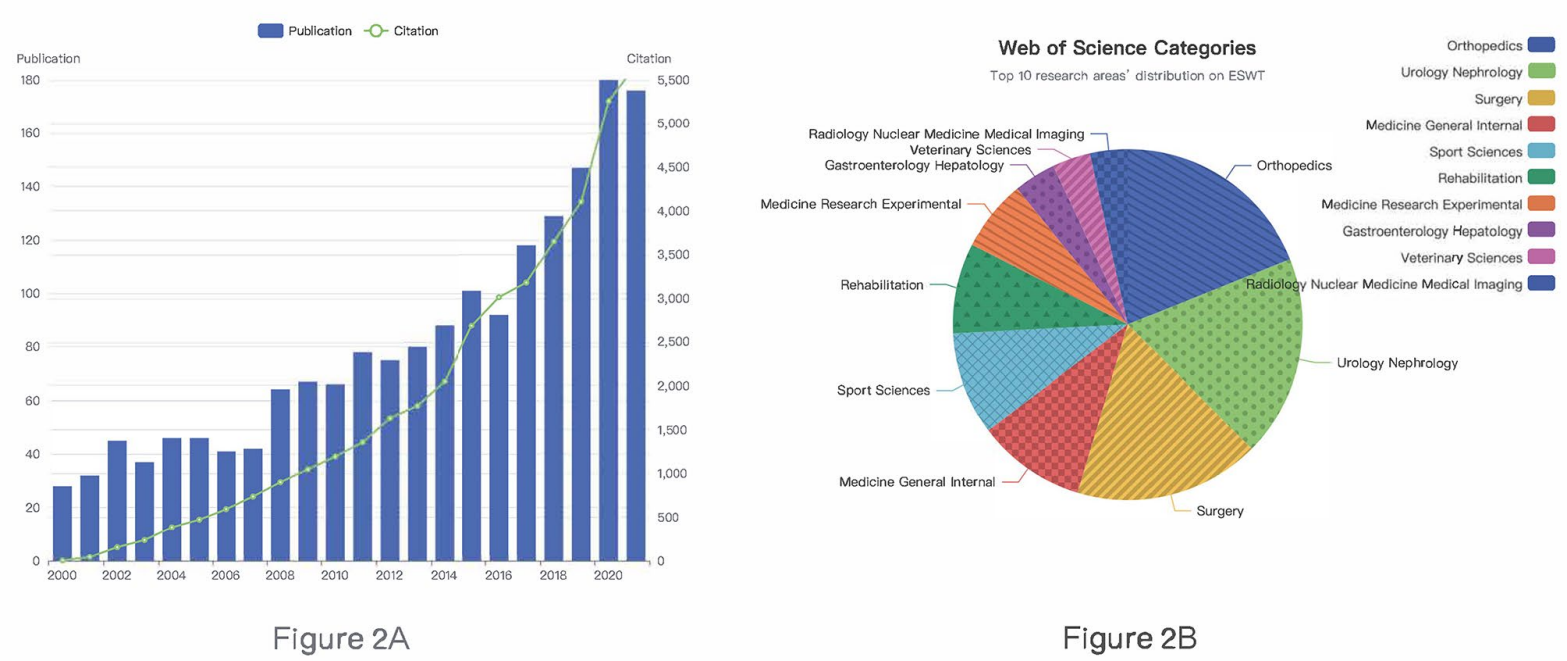
**(A)** Annual publication and citation number of the literature from 2000 to 2021. **(B)** The top ten categories’ distribution on ESWT

Initially, 2231 publications were retrieved from the Web of Science Core Collection for the period 2000–2021 which were associated with extracorporeal shock wave therapy. Then we included 1774 publications according to the screening criteria (Fig. 1). According to Fig. 2A, although there were some fluctuations in the publication data, the overall trend was upward. The number of sources increased from 28 to 2000 to 176 in 2021. In Fig. 2B, the topmost research fields were Orthopedics (315, 17.8%), Urology Nephrology (309, 17.4%), Surgery (286, 16.1%), Medicine General Internal (168, 9.47%), Sport Sciences (157, 8.85%), symbolizing their core positions.

### Analysis of countries and institutions

**Fig. 3:**
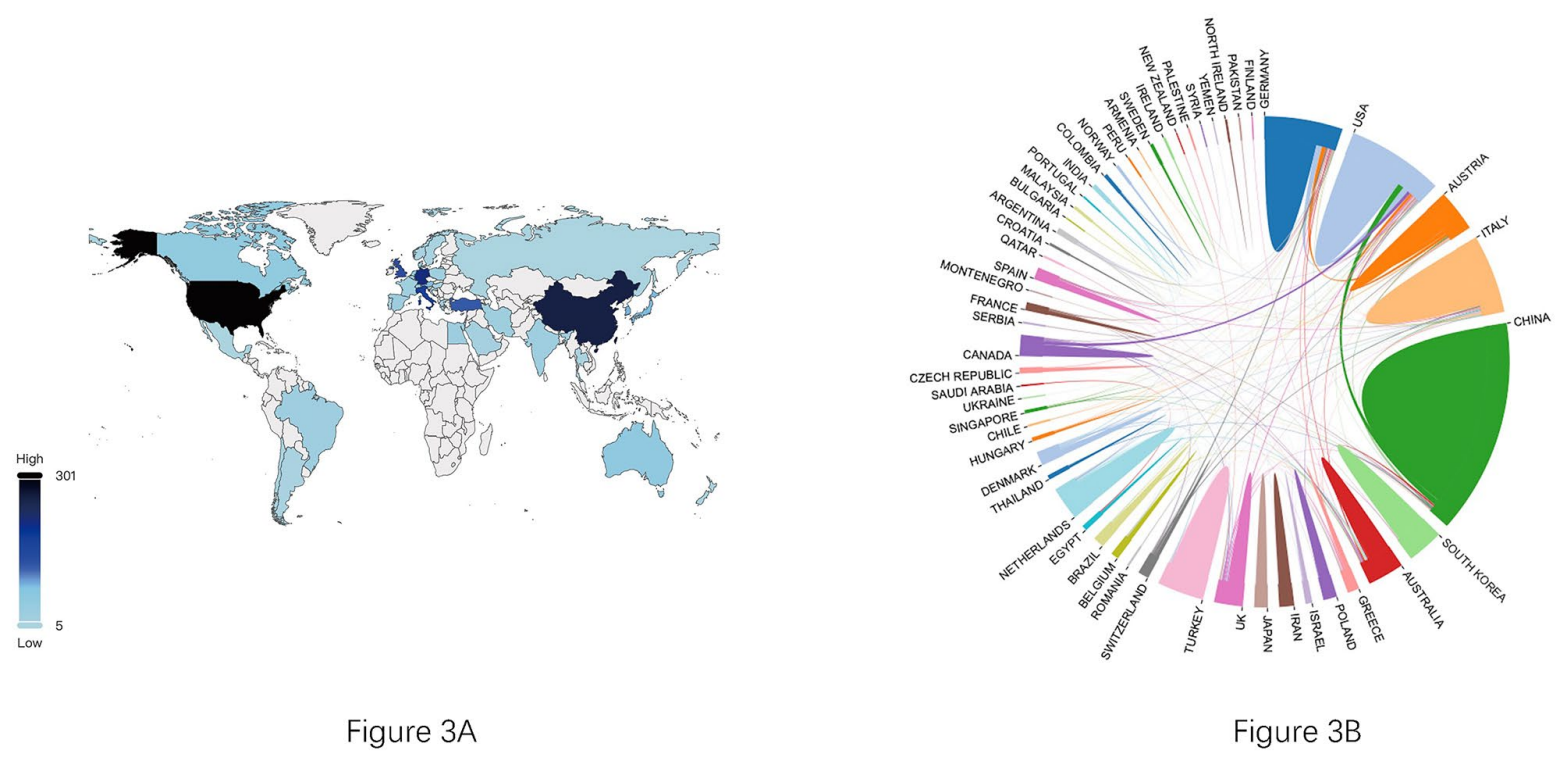
**(A)** The distribution of published publications by countries/regions in a world map. **(B)** The collaboration between two countries/regions. The size of each sector is proportional to the number of publications, while each line is directly proportional to the degree of scientific cooperation between the two countries

According to the search results, 71 countries/regions contributed all the literature that meets the criteria. In Table 1, we can see the top 10 countries/regions with the most publications in the ESWT field, and their contributions accounted for 89% of the total. Among the most scientific nations in the world, the United States (301, 16.967%) produces the most publications, then followed by China (256, 14.431%), and Germany (209, 11.781%). Figure 3 A can present a visual representation of the distribution of published papers around the world, which can reveal that some Nordic countries contribute significantly in addition to the United States and China. In the collaborative network (Fig. 3B), the thickness of the line between each sector represents the degree of scientific cooperation between countries, and the spans of lines between China and the United States are the widest, indicating the large partnership community between the two countries for ESWT research.

**Table 1:** Top 10 countries (or regions) contributed on ESWT.

Countries/Regions	Record Count	% of 1,774
USA	301	16.967
PEOPLES R CHINA	256	14.431
GERMANY	209	11.781
ITALY	171	9.639
TAIWAN	154	8.681
TURKEY	121	6.821
ENGLAND	120	6.764
SOUTH KOREA	85	4.791
JAPAN	83	4.679
AUSTRIA	82	4.622

**Fig. 4:**
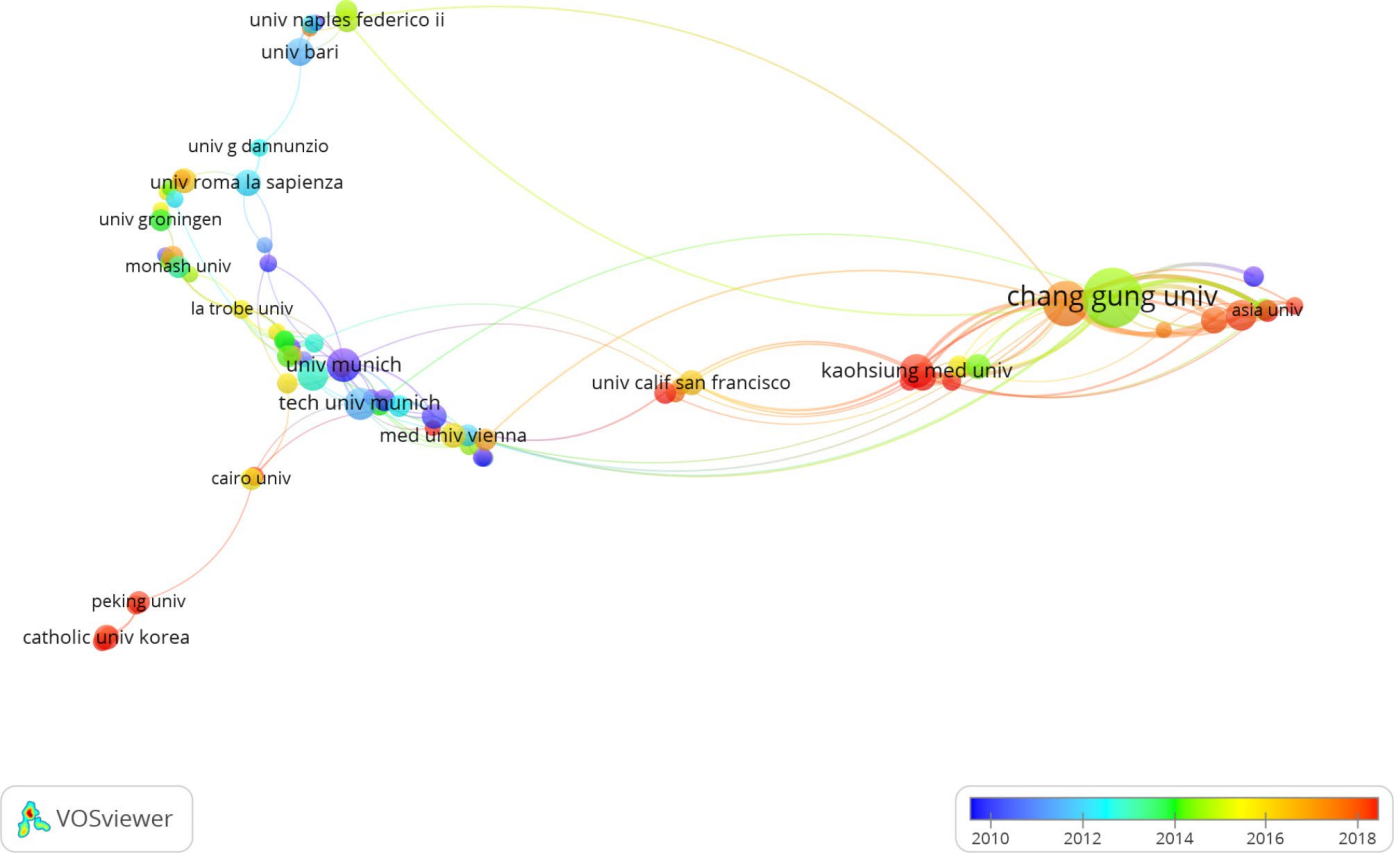
Co-authorship overlay visualization map of institutions. The size of each circle represents the number of publications of the related institution. The thickness of the line represents the closeness between two institutions. Dark blue and light blue dots stand for earlier, while orange and red dots stand for the last five years

The top 3 institutions contributing to the ESWT research are Chang Gung University (n = 123), Kaohsiung Chang Gung Memorial Hospital (n = 120), and Kaohsiung Medical University (n = 50), reflecting the outstanding progress conducted by the Taiwan region in ESWT study (Table 2). Furthermore, we used VOSviewer software to analyze the collaborative visualization network among these institutions (Fig. 4). The results can reveal that there was close cooperation among several research institutes in the Taiwan region, such as Chang Gung University, Kaohsiung Medical University, China medical university Taiwan, and National Taiwan University. Thus, it can be seen that Major Universities in Taiwan did attach great importance to ESWT.

**Table 2:** The main institutions contributing to publications on ESWT research

Rank	Institution	Article counts	Total number of citations	Average number of citations	Total number of first author	Total number of first author citations	Average number of first author citations
1	Chang Gung University	123	1353	11	27	410	15.19
2	Kaohsiung Chang Gung Memorial Hospital	120	519	4.33	25	127	5.08
3	Kaohsiung Medical University	50	209	4.18	7	19	2.71
4	University of Bari	34	391	11.5	11	111	10.09
5	Chang Gung Memorial Hospital	25	653	26.12	10	253	25.3
6	Tech University Munich	25	390	15.6	6	116	19.33
7	Tohoku University	25	185	7.4	10	64	6.4
8	Catholic University Korea	24	66	2.75	10	27	2.7
9	University Groningen	23	189	8.22	10	125	12.5
10	University Munich	23	387	16.83	10	120	12

### Analysis of journals

Since 2000, a total of 471 journals have published articles on ESWT. We identified the 10 most-cited journals in the past 22 years, which published 145 papers, accounting for 8.17% of total publications (Table 3). Therefore, paying attention to the publication of these central journals benefits keeping up with the latest trends. *JOURNAL OF ORTHOPAEDIC RESEARCH*, *AMERICAN JOURNAL OF SPORTS MEDICINE*, and *JOURNAL OF SURGICAL RESEARCH* are the top three most-cited journals. *EUROPEAN UROLOGY*, *AMERICAN JOURNAL OF SPORTS MEDICINE*, and *JOURNAL OF SEXUAL MEDICINE* are the three highest-IFs journals. *JOURNAL OF ORTHOPAEDIC RESEARCH*, *AMERICAN JOURNAL OF SPORTS MEDICINE*, *EUROPEAN UROLOGY* and *WOUND REPAIR AND REGENERATION* are classified as Q1 according to journal Citation reporting standards and are an important source of knowledge for ESWT.

**Table 3:** The most cited journals contributing to publications on ESWT research

Journals	Total Number of Citations	Average Number of Citations	Article counts	IFs	Quartile in category [2020]	H-index
JOURNAL OF ORTHOPAEDIC RESEARCH	308	30.8	10	3.494	Q1	19
AMERICAN JOURNAL OF SPORTS MEDICINE	271	15.94	17	6.202	Q1	31
JOURNAL OF SURGICAL RESEARCH	259	21.58	12	2.192	Q3	19
ARCHIVES OF ORTHOPAEDIC AND TRAUMA SURGERY	246	13.67	18	3.067	Q2	18
ULTRASOUND IN MEDICINE AND BIOLOGY	195	7.5	26	2.998	Q2	24
FOOT & ANKLE INTERNATIONAL	186	9.3	20	2.827	Q2	16
EUROPEAN UROLOGY	172	11.47	15	20.096	Q1	56
WOUND REPAIR AND REGENERATION	157	26.17	6	3.617	Q1	13
JOURNAL OF SEXUAL MEDICINE	151	13.73	11	3.802	Q2	20
JOURNAL OF ORTHOPAEDIC SURGERY AND RESEARCH	123	12.3	10	2.359	Q3	16

### Analysis of research hotspots

Hotspot analysis of keywords is the most essential step in the bibliometric analysis. According to the keywords extracted from the literature, the previous hotspots were summarized intuitively and the latest hotspots may be predicted according to the recent hot keywords. Using high-frequency keywords to illuminate the research hotspots in a discipline can efficiently decide the research hotspots and other important issues.

Bibliographic Items Co-occurrence Matrix Builder (BICOMB) is a software used to select high frequency keywords for cluster analysis and construct the keyword-source article matrix and keyword co-occurrence matrix [14]. Among the 1774 ESWT-related publications, a total of 3195 keywords were extracted by the BICOMB software. Then we defined the keywords whose frequency was at least 13 times or more as the high-frequency keywords. After removing the repeated words, as a consequence, 30 aimed keywords are targeted (Table 4). Next, we excluded the keywords without actual referential meanings, concluding the result that the top 5 most frequent words are lithotripsy, erectile dysfunction, plantar fasciitis, pain, and rehabilitation. The next step is to generate a binary co-occurrence matrix from these keywords by BICOMB in txt format. Successively, gCLUTO was used for biclustering analysis, and the mountain and heat maps were drawn based on this. Additionally, VOSviewer was used for visualization analysis of the keywords that co-occurred at least 5 times or more.

**Table 4:** The most frequent keywords in the ESWT research

Rank	Keywords	Frequency	Percentage%	Cumulative Percentage%
1	extracorporeal shock wave therapy	558	9.1057	9.1057
2	ESWT	109	1.7787	10.8845
3	lithotripsy	79	1.2892	12.1736
4	extracorporeal shock wave lithotripsy	78	1.2728	13.4465
5	erectile dysfunction	58	0.9465	14.393
6	plantar fasciitis	57	0.9302	15.3231
7	shock wave	54	0.8812	16.2043
8	pain	49	0.7996	17.0039
9	rehabilitation	46	0.7507	17.7546
10	urolithiasis	45	0.7343	18.4889
11	tendinopathy	41	0.6691	19.158
12	meta-analysis	39	0.6364	19.7944
13	angiogenesis	39	0.6364	20.4308
14	treatment	33	0.5385	20.9693
15	radial extracorporeal shock wave therapy	33	0.5385	21.5078
16	ESWL	31	0.5059	22.0137
17	osteoarthritis	28	0.4569	22.4706
18	lateral epicondylitis	26	0.4243	22.8949
19	chronic pancreatitis	26	0.4243	23.3192
20	shoulder	25	0.408	23.7272
21	spasticity	21	0.3427	24.0698
22	shock wave therapy	21	0.3427	24.4125
23	Peyronie′s disease	18	0.2937	24.7063
24	stroke	17	0.2774	24.9837
25	systematic review	16	0.2611	25.2448
26	Knee	14	0.2285	25.4732
27	ultrasonography	13	0.2121	25.6854
28	inflammation	13	0.2121	25.8975
29	horse	13	0.2121	26.1097
30	kidney calculi	13	0.2121	26.3218

**Fig. 5:**
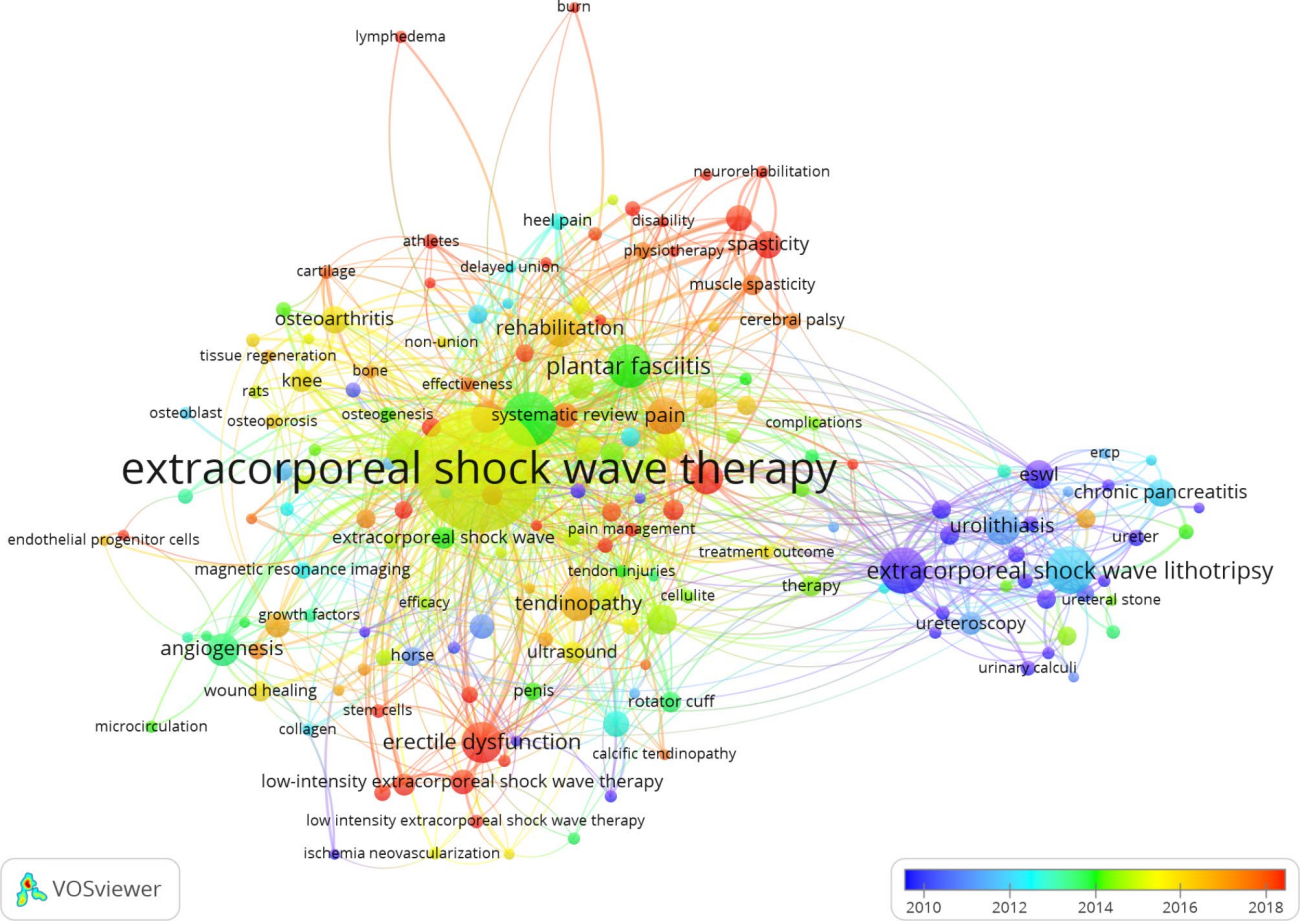
Keywords co-occurrence overlay visualization map. The description is the same as in Fig. 4

Keywords co-occurrence analysis is to count the frequency of the occurrence of keywords in the included literature and analyze the internal relationships among them. The size of the dots represents the frequency of keyword occurrences, and the thickness of the lines between the dots represents relevance between 2 words. The overlay timeline in the lower right corner (Fig. 5) shows the published year in which the keywords appeared. Dark blue and light blue dots represent earlier research hotspots, while orange and red ones represent research hotspots in the last five years. Then we set the frequency of keyword occurrence to at least 9 times and got Fig. 5. ESWL (extracorporeal shock wave lithotripsy), urolithiasis, renal calculi, and ureteral calculi were the early research directions, while in recent years, the research directions are lymphedema, delayed union, cellulite, burn, and diabetic foot ulcer, etc. (Fig. 5).

**Fig. 6:**
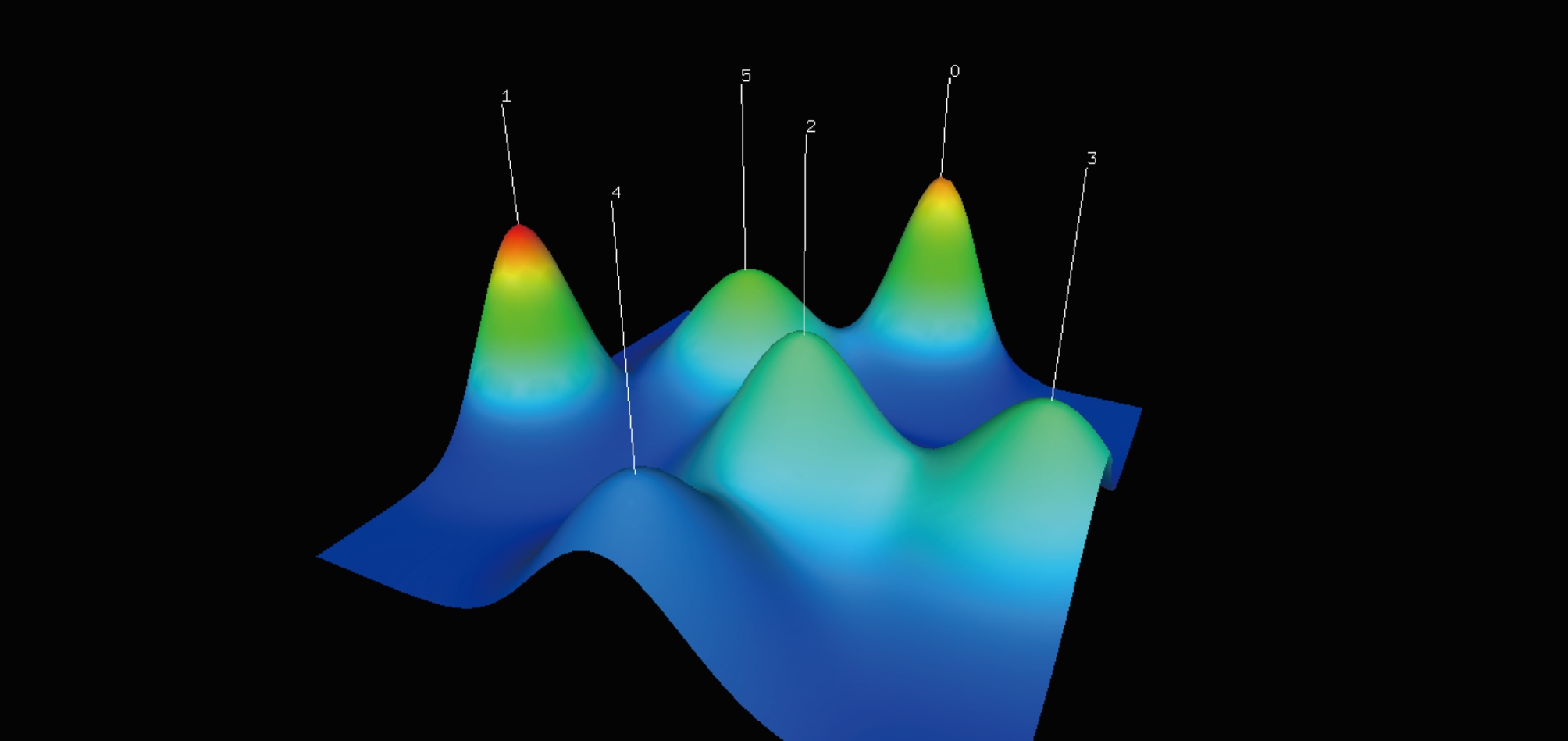
The visualization mountain map. The volume of the hill is proportional to the intra-class similarity, and the height is proportional to the number of keywords contained within the cluster. The color of the mountain is related to the standard deviation within the class. Red means low standard deviation, and blue means high standard deviation

Currently, gCLUTO software includes two visualization schemes: visualization matrix map and visualization mountain map. As shown in Fig. 6, there are 6 colorful visualized mountains. The volume, height, and color of each mountain all give pieces of evidence about the corresponding cluster. The volume of the hill is proportional to the intra-class similarity, and the height is proportional to the number of keywords contained within the cluster. The color of the mountain is related to the standard deviation within the class. Red means low standard deviation, and blue means high standard deviation. Also, it is meaningful to observe the color at the top of the hill, while the other areas are mixed just to make the color smooth over.

**Fig. 7:**
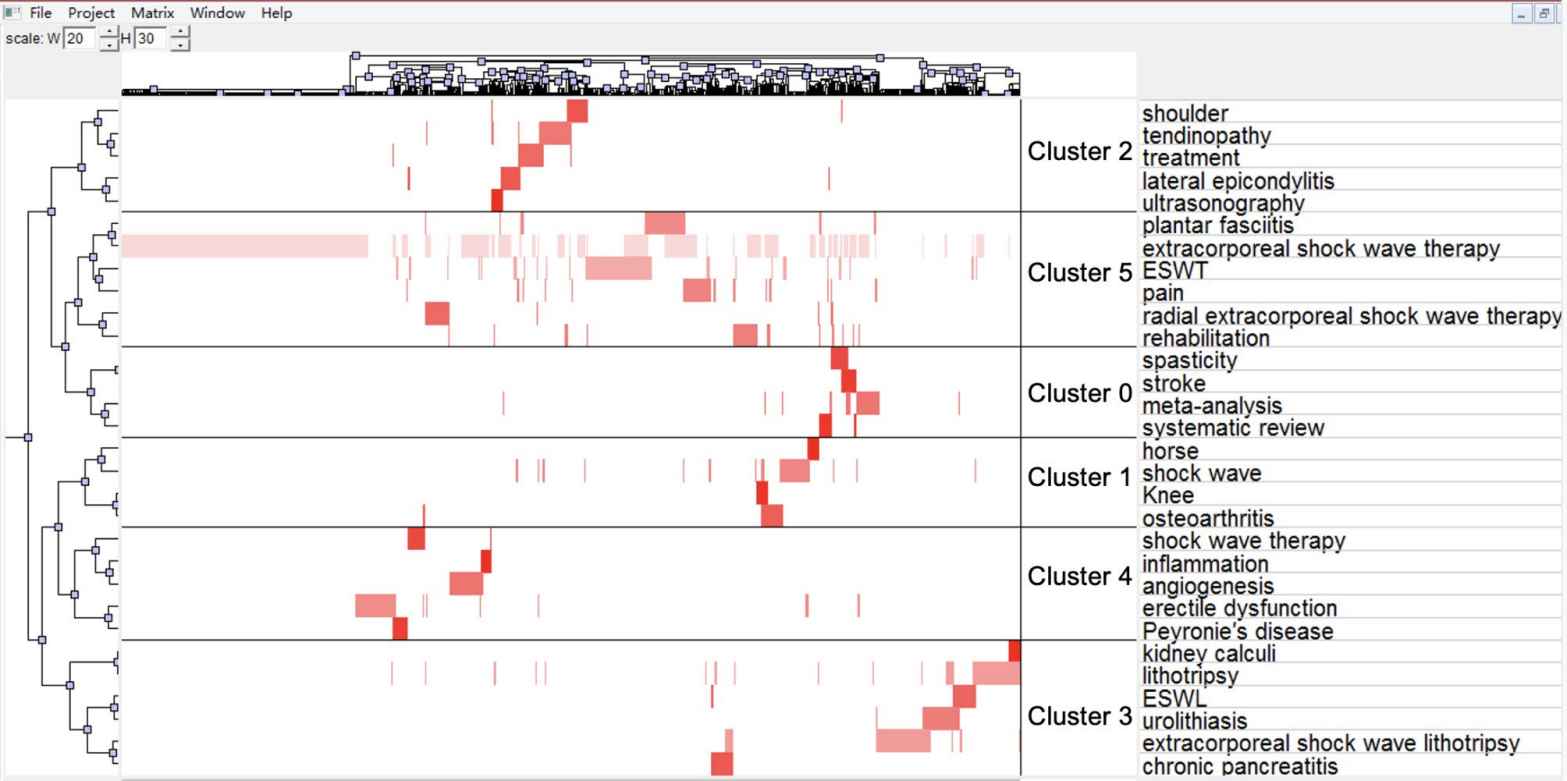
The visualization matrix map. The colors represent the values in the original data matrix. While white symbolizes near-zero values, a deepening red implies larger values

In Fig. 7 (the visualization matrix map), the colors represent the values in the original data matrix. While white symbolizes near-zero values, a deepening red implies larger values, and the white colour indicated negative value. The rows of the matrix are rearranged so that the rows of the same class are together with the black horizontal lines separating the classes. Next, we conducted a clustering-analysis on 30 high-frequency keywords in Table 4 and confirmed 6 hotspots in the ESWT research field:

Cluster0: The effects of ESWT on muscle spasticity.

Cluster1: The application of ESWT in osteoarthritis (OA).

Cluster2: Therapeutic effect of ESWT on tendon diseases.

Cluster3: Early application of ESWT/ESWL in urolithiasis.

Cluster4: The Role of angiogenesis in ESWT and the efficiency of ESWT for penile disease.

Cluster5: The Special value of radial extracorporeal shock wave therapy (rESWT).

## Discussion

In this study, we provide a comprehensive description of bibliometrics indicators in the research field of ESWT. 1774 articles were analyzed that met the criteria for ESWT publications from January 1, 2000, to December 31, 2021. Most studies were conducted in the United States and China, followed by Germany, Italy, and the Taiwan region, Furthermore, with the most cooperation in the United States and China. The most published research institutions are Chang Gung University, Kaohsiung Chang Gung Memorial Hospital, and Kaohsiung Medical University. *JOURNAL OF ORTHOPAEDIC RESEARCH*, *AMERICAN JOURNAL OF SPORTS MEDICINE*, and *JOURNAL OF SURGICAL RESEARCH* are the top three most-cited journals.

We identified six research hotspots through keyword cluster analysis, which are Cluster0 ~ Cluster5. Cluster0: The effects of ESWT on muscle spasticity; Cluster1: The application of ESWT in osteoarthritis (OA); Cluster2: Therapeutic effect of ESWT on tendon diseases; Cluster3: Early application of ESWT/ESWL in urolithiasis; Cluster4: The Role of angiogenesis in ESWT and the efficiency of ESWT for penile disease; Cluster5: The Special value of radial extracorporeal shock wave therapy (rESWT). Therefore, current research status, trends and future challenges related to these six research hotspots were systematically described in our study.

### Cluster0: The effects of ESWT on muscle spasticity

Spasticity is neurological damage caused by upper motor neuron (UMN) syndrome [15]. In recent years, mounting evidence that demonstrates ESWT is a secure and efficient alternative for reducing muscle spasticity has been collected [16, 17]. Although the mechanism behind the effects of ESWT in spasticity remains uncertain, pertinent studies have suggested the following possibilities: inducing NO production [18, 19], reducing motor neuron excitability [20], dysfunction in neuromuscular transmission [21], affecting rheological properties, and appearing fibrosis of chronic hypertonic muscles [22].

Another disease that commonly causes spasticity is cerebral palsy (CP). In a clinical trial [23], the scientists found after a shockwave stimulation, a significant change was definitely observed in the MAS, containing an growth in passive range of motion (PROM), and an increase in the contact plantar surface area of the treated limb in patients with spastic equinus foot. Some scientists even concluded that the therapeutic effect of CP was positively correlated with the sessions of ESWT [24].

### Cluster1: the application of ESWT in osteoarthritis (OA)

OA is quite common in the elderly, with more than 40% incidence in people aged 65 and older [25]. The knee and hip are the two main joints involved. The definition of OA is a metabolic abnormality of joint tissue, followed by anatomical and physiological disorders [26]. The main symptoms are pain and stiffness in the affected joints [27]. It was once thought that the pathogenesis of osteoarthritis was merely a cartilage degeneration disease, whereas now the findings discovered that it is a complex joint disorder, related to cartilage, subchondral bone, synovium, etc. [28].

In very recent years, ESWT has become a novel conservative treatment for osteoarthritis. The advantanges are as follows: (1) Increase chondrocyte activity, reduce cartilage fissuring, and inhibit chondrocyte apoptosis [29]. (2) Promote osteocyte proliferation and make better tissue distributions among cortical bone, cancellous bone, and fibrous tissue [30, 31]. (3) Relieve the patient’s pain [32]. (4) Improve the patient’s motor function [33]. Previous studies have discovered that ESWT may play a role not only in the early stages of OA, but throughout the entire course of the disease [34]. Additionally, ESWT has been shown to alleviate pain associated with OA [32, 35] and may do so by suppressing the production of Calcitonin Gene-Related Peptide (CGRP) from dorsal root ganglia (DRG), which in turn reduces the transmission of pain through sensory nerve fibers. [36].

### Cluster2: therapeutic effect of ESWT on tendon diseases

Tendinopathy is a term used to describe a condition that affects tendons, which are fibrous tissues that connect muscles to bones. It is characterized by pain and reduced function in the affected tendon, and is often the result of overuse or degeneration due to aging [37]. Tendinopathy can occur in any tendon in the body, but is most commonly seen in tendons in the shoulder, elbow, wrist, hip, knee, and ankle. The symptoms of tendinopathy can vary depending on the location and severity of the condition, but typically include pain, tenderness, swelling, and reduced strength and flexibility in the affected area [37].

Some studies have shown that ESWT has good efficacy in the treatment of tendinopathy. Experimental studies have demonstrated that ESWT can promote regeneration of tendinous tissue, reduce inflammation, and improve tendinous function [38, 39]. At first, it was thought that ESWT could treat calcifying tendinitis because “lime” could be flushed out of the tendon, just like a shockwave shattering a kidney stone and passing it through the ureter. However, it was proved that ESWT’s treatment effect is on tendon repair [40].

Several clinical trials have also proven the effectiveness of ESWT in the treatment of tendinopathy [41]. The Clinical trial results show that most patients significantly reduce pain and improve muscle function and range of motion after receiving ESWT treatment [42].

Some comparative studies have also demonstrated the advantage of ESWT in the treatment of tendinopathy. These studies typically compare ESWT with other treatments, such as drug therapy and surgical treatment, and find that ESWT has better efficacy and fewer side effects [43, 44]. For lateral epicondylitis, routine injection therapy includes steroid injections, botulinum toxin A (BoNT-A), autologous whole blood, platelet-rich plasma (PRP), and dextrose prolotherapy (DPT) [45]. Additionally, ESWT shows superiority for pain relief and grip strength recovery in lateral epicondylitis [4].

### Cluster3: early application of ESWT/ESWL in urolithiasis

Extracorporeal Shock Wave Therapy (ESWT) is a medical treatment that involves the delivery of shock waves to the affected area of the body. The therapy was originally developed as a means of breaking up kidney stones, a procedure known as extracorporeal shock wave lithotripsy (ESWL) [46, 47]. Over time, researchers and medical professionals discovered that the shock waves produced by ESWL had other therapeutic effects, including the promotion of healing and the reduction of pain and inflammation. This led to the development of ESWT as a standalone treatment for a variety of conditions, including tendinopathy, plantar fasciitis, and calcifying tendinitis, among others. In ESWT, shock waves are generated outside the body and are focused on the target tissue through the skin and underlying tissues using a specialized probe. The shock waves promote healing by stimulating the body’s natural repair processes and reducing pain and inflammation.

However, it is conspicuous that the use of ESWL has declined in recent years due to the occasional complications [48], which include infection [49], renal subcapsular hematoma [50], related organs trauma [51], chronic pancreatitis [52], and urinary tract obstruction due to stone fragments [49]. Therefore, the most important preventive measures are to recognize the limitations of ESWL, adopt alternative therapies, correct existing renal or systemic diseases, treat urinary system infections, and use preventive antibiotics [49]. In addition, the use of slower pulse rates, ramping strategies, and adequate coupling of the shock wave head can significantly increase the efficacy and safety of ESWL [53].

### Cluster4: the role of angiogenesis in ESWT and the efficiency of ESWT for penile disease

A number of studies have shown that ESWT exerts positive effects on cell proliferation and angiogenesis [54–57]. Vascular endothelial growth factor (VEGF) was significantly increased in tissue samples of ESWT-treated rat models [58]. In Balsoli ‘s study [59], human foreskin fibroblast cells were exposed to shock waves (100 pulses, 0.19 mJ/mm^2^, 3 Hz) for 5 minutes. The results showed that cell proliferation, production of reactive oxygen species (ROS), and ATP release increased significantly.

Additionally, Since Vardi et al. revolutionarily discovered that ESWT could treat erectile dysfunction (ED) [60], more and more studies have found that ESWT plays a certain role in andrology diseases, including Peyronie’s disease (PD), benign prostatic hyperplasia (BPH), and chronic pelvic pain syndrome (CPPS). Studies [61] have shown that ESWT for ED seems to improve cavernosal function possibly through stimulation of mechanosensory, remodeling of erectile tissue, stimulating the activation of angiogenesis, recruitment, and activation of progenitor cells, improving microcirculation, nerve regeneration, and reducing inflammatory reactions. In addition, studies have shown that low-intensity ESWT (Li-ESWT) can be effective for ED patients up to two years after treatment [62].

In a meta-analysis [63], ESWT significantly reduced the proportion of penile plaques and relieving pain in PD patients. Moreover, in Zhang’s study [64], ESWT was testified to be effective in BPH patients who did not respond to a drug or surgical treatment. In 3 months after treatment in patients with maximum urinary flow rate increases, the bladder residual urine volume decreases. In the CPPS study [65], the experimental group treated with perineal ESWT (3000 pulses, 0.25 mJ/mm^2^, 4 Hz) weekly experienced a reduction in refractory pain and a significant improvement in quality of life after 6 weeks of treatment.

### Cluster5: the special value of radial extracorporeal shock wave therapy (rESWT)

Two main types of generators can create shock waves: focused ESWT (fESWT), and radial ESWT (rESWT). Between these 2 different treatments, fESWT is more intense within a targeted area, while rESWT has a more widespread but superficial region of action [66]. Therefore, rESWT is considered a less invasive tool and is more appropriate for conservative therapy [67]. RESWT, as a new treatment in the field of rehabilitation medicine in recent years, not only has a short treatment time, and a long treatment interval, but also has a broad indication. Currently, rESWT is widely applied in orthopedic diseases. At the same time, diabetic foot ulcer [68], primary dysmenorrhea [69], heterotopic ossification [70], and cellulite [71] also have a good clinical effect after treated with rESWT. The specific features (probe, frequency, intensity, points) of rESWT in each disease need individual consideration and comprehensive evaluation, which is also a major focus and difficulty in the field of rehabilitation medicine.

### Strengths and limitations

This study has strengths. This bibliometric study is the first of its kind, to our knowledge, to identify and characterize ESWT-related articles across all journals in the ISI Web of Science SCIE. This study aims to provide insight into the current research trends on ESWT worldwide through the use of bibliometric and visualization analysis. However, it’s essential to acknowledge some limitations in the interpretation of our findings.

Firstly, database disparities can pose a challenge in bibliometric analysis. Databases such as WoS, PubMed, Embase, and Cochrane Library all have different publications, and our study may have missed some articles due to database bias. Our literature search was conducted using the SCIE databases, and we only included English-language studies based on WoS, which may result in language bias by excluding non-English publications.

Secondly, there may be a discrepancy between the results of the bibliometric analysis and the real-world study conditions. For instance, some recently published high-quality studies may not have high citation frequencies and therefore may not be reflected in our results.

## Conclusion

With the gradual increase of research on ESWT, it demonstrates the application of ESWT is more and more extensive/comprehensive, such in musculoskeletal disease, bone delay union, neurological injury, andrology disorders, lymphedema, and so on. In addition, the mechanism is not destructive damage, as initially thought, but a restorative treatment. In this article, we used bibliometrics to summarize the knowledge related to ESWT. The 6 hotspots are the focus of our research. Among them, the research on ESWT in spasticity and osteoarthritis is relatively mature, while the research in promoting angiogenesis is less mature. Furthermore, delayed union, cellulite, burn and diabetic foot ulcer are also popular in shockwave research, and may navigate the future direction of scientific study. This study provide a more intuitive overview for researchers, and more scholars are expected to participate in the research field of ESWT.

## Data Availability

Not applicable.

## List of Abbreviations

ESWT: Extracorporeal shock wave therapy
WoSCC: Web of Science Core Collection
BICOMB: Bibliographic item co-occurrence matrix builder
gCLUTO: Graphical clustering toolkit
ESWL: Extracorporeal shock wave lithotripsy
OA: Osteoarthritis
rESWT: Radial extracorporeal shock wave therapy
fESWT: Focused extracorporeal shock wave therapy
UMN: Upper motor neuron
MAS: Modified Ashworth Scale
FDS: Flexor digitorum superficialis
FCR: Flexor carpi radialis
MTS: Modified Tardieu Scale
VAS: Visual analog scale
CP: Cerebral palsy
PROM: Passive range of motion
ACLT: Anterior cruciate ligament transection
CGRP: Calcitonin gene related peptide
DRG: Dorsal root ganglia
BoNT-A: Botulinum toxin A
PRP: Platelet-rich plasma
DPT: Dextrose prolotherapy
VEGF: Vascular endothelial growth factor
ROS: Reactive oxygen species
ED: Erectile dysfunction
PD: Peyronie’s disease
BPH: Benign prostatic hyperplasia
CPPS: Chronic pelvic pain syndrome
Li-ESWT: Low-intensity ESWT
